# An African perspective on the genetic diversity of *Toxoplasma gondii*: A systematic review

**DOI:** 10.1017/S0031182023000252

**Published:** 2023-06

**Authors:** Ilze du Plooy, Malitaba Mlangeni, Riann Christian, Ana M. Tsotetsi-Khambule

**Affiliations:** Department of Life and Consumer Sciences, College of Agriculture and Environmental Sciences, University of South Africa, Florida 1709, South Africa

**Keywords:** Africa, genetic diversity, genotype, ToxoDB, *Toxoplasma gondii*, toxoplasmosis

## Abstract

The study of *Toxoplasma gondii* genotypes is beneficial for detecting strains linked to increased disease severity and uncovering the processes involved in the transmission and distribution of this zoonotic parasite. A systematic review of literature was conducted to investigate the present status of *T. gondii* genetic diversity in African countries and among host species on the continent. Data from the results in the included studies were sorted, reviewed and descriptively analysed using tables, graphs and maps. Results indicate that there is a relative amount of genetic diversity with a clear difference in the population structure between geographical regions and the propensity for unique and regional genotypes to be predominant in tropical rainforest biomes, near the equator. From a clinical perspective, connections between specific *T. gondii* genotypes and disease manifestations were found. Theories are outlined on the dissemination of African *T. gondii* genotypes to other continents. The overrepresentation of samples from one geographical area and dissimilar genotyping methodologies creates challenges when concluding on the genetic diversity of *T. gondii* in Africa. The need for uniform genotyping methods with a continent-wide sampling of an extensive host range involving humans, domestic animals and wildlife is emphasized.

## Graphical abstract

**Figure:**
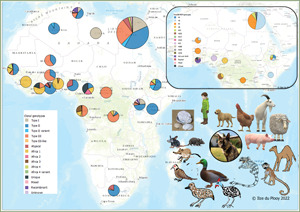

## Introduction

*Toxoplasma gondii* is an obligate intracellular apicomplexan protozoan parasite causing toxoplasmosis, a priority zoonotic disease of One Health importance. Countless species of warm-blooded vertebrates including humans are susceptible to *Toxoplasma* infection (Kistiah *et al*., 2012; Galal *et al*., 2018; Despommier *et al*., 2019; Shah and Khan, 2019; Al-Malki, 2021; Simpson *et al*., 2021). There have also been rare occurrences of *T. gondii* being detected in reptiles and molluscs (Nasiri *et al*., 2016; Ghozzi *et al*., 2017; Feitosa *et al*., 2018). The lifecycle involves asexual cycles in the intermediate hosts (warm-blooded vertebrates) and both asexual and sexual cycles in the definitive host (felines) (Dubey, 1996). Infectious stages of the organism include tachyzoites (invasive form), bradyzoites (present in tissue cysts) and oocysts containing sporozoites (shed in feline feces) (Al-Malki, 2021). Infection can occur by consuming tissue cysts in raw or undercooked meat, ingesting oocysts present in the environment, contaminated food or water, ingestion of circulating tachyzoites in raw goats' milk, tachyzoite infection *via* organ or bone marrow transplants or blood transfusions and congenital transmission of tachyzoites crossing the placenta (Dubey, 1996; Khan *et al*., 2005; Robert-Gangneux and Dardé, 2012; Hammond-Aryee *et al*., 2014; Despommier *et al*., 2019). According to Galal *et al*. (2018), several factors can influence the prevalence and severity of disease such as the method of infection, the inoculum dose, recurrent infections, age, ethnicity, immune status, the screening system used, the presence of coinfections and the genotypes of the strains involved.

Distribution is worldwide with a seroprevalence rate of 25.7%, which is estimated to be higher in Africa compared to other continents (Molan *et al*., 2019). This zoonotic parasite has classically been grouped into 3 archetypal clonal lineages, types I, II and III, which emerged from a common ancestor ±10 000 years ago (Su *et al*., 2003; Dardé, 2008). In most geographical regions around the world, clonal lineages predominate, with each region having a higher prevalence of a particular clonal or regional genotype with relatively low levels of diversity (Dardé, 2008; Su *et al*., 2012; Shwab *et al*., 2018). In contrast, Central/South America has an abundance of genetic diversity with less common genotypes and genetically distinct isolates in different regions (Dardé, 2008; Su *et al*., 2012; Shwab *et al*., 2018; Galal *et al*., 2019*a*).

The expansion of the 3 classic clonal lineages and their abundance across the world has been linked to the simultaneous adaptation of direct oral infectivity traits in combination with the adoption of cats as companion animals and the advent of agricultural animal domestication (Su *et al*., 2003; Sibley *et al*., 2009; Shwab *et al*., 2018). The isolates could thus replicate asexually through successive hosts within population groups and bypass sexual recombination creating an expansion on a clonal level.

Identification of *T. gondii* genotypes is useful in epidemiological studies of toxoplasmosis for the detection of specific strains that can be linked to higher virulence (Khan *et al*., 2005; Dardé, 2008; Liu *et al*., 2015). Developed genotyping methods include microsatellite (MS) analysis, multilocus sequence typing (MLST), restriction fragment-length polymorphism PCR (PCR-RFLP), random amplified polymorphic DNA-PCR and high-resolution melting analysis (Liu *et al*., 2015). Multilocus nested-PCR-RFLP genotyping using 10 molecular markers has aided in the discovery of 189 different genotypes currently listed on the *Toxoplasma* genome database, http://ToxoDB.org (Liu *et al*., 2015; Harb and Roos, 2020). Clonal lineages have also been defined by using 15 polymorphic MS markers that are distributed across 11 different chromosomes (Ajzenberg *et al*., 2010). Classifications of clonal lineages include archetypal lineage (types I, II and III), variants of archetypal lineages, local or regional clonal lineages (*Amazonian*, *Africa 1–4*, *Caribbean 1–3*, *Chinese 1* and *Guiana*) and unique genotypes or atypical genotypes (Galal *et al*., 2019*a*). Multilocus sequence typing assays have revealed clusters of 16 haplogroups belonging to 6 ancestral clades with most strains grouped into this clonal structure (Su *et al*., 2012). Genetic analyses of *T. gondii* isolates with multiple methods have shown that there are correspondences between whole-genome sequencing, MLST, PCR-RFLP and 15 MS markers (Su *et al*., 2012; Shwab *et al*., 2014; Galal *et al*., 2019*a*). These studies, which included a total of 1457 isolates originating from all continents except Antarctica, have made correlations between conventional genotype designations and ToxoDB PCR-RFLP defined genotypes (Shwab *et al*., 2014). Designations for some of the lineages derived from the tested isolates which are also of importance in this review include ToxoDB#1 (type II clonal), ToxoDB#3 (type II variant), ToxoDB#2 (type III), ToxoDB#10 (type I), ToxoDB#6 (*Africa 1*), ToxoDB#203 (*Africa 3*), ToxoDB#137 and #20 (*Africa 4*) (Shwab *et al*., 2014; Galal *et al*., 2019*a*).

Different genotypes of *T. gondii* have been linked to disease severity with a variance in virulence among the genotypes (Dardé, 2008). Infection with a particular strain is reliant on the geographical origin where the infection was acquired and virulent strains containing type I or atypical alleles have been found to be more pathogenic and more likely to cause severe disease due to the type of immune response elicited inside the host (Xiao and Yolken, 2015). Despite evidence that *T. gondii* genotype might be associated with disease severity, there is a deficiency in investigations focusing on the topic, especially in Africa (Galal *et al*., 2018). Knowledge of the population structure of *T. gondii* can aid in discovering the impact of genotypes on disease manifestations, what geographical determinants influence the genotype and contribute to the development of new diagnosis, treatment and vaccine strategies (Sharif *et al*., 2017; Lachkhem *et al*., 2021). This study, therefore, provides a summary of the genotypes of *T. gondii* that have been identified thus far in African countries and an evaluation on the prevalence of genotypes in the different countries and among host species.

## Materials and methods

### Search strategy

This review followed the preferred reporting items for systematic reviews guidelines (Page *et al*., 2021*a*, 2021*b*). A systematic review of literature published until April 2022 was conducted to explore the genetic diversity of *T. gondii* in African countries. Searches were conducted on 6 online databases which included Europe PMC, PubMed, ScienceDirect, Scopus, Google Scholar and Dimensions AI. Boolean operators ‘AND’ and ‘OR’ were used for the searches in each database. Where possible, searches were limited to the title and abstract together with keywords or text words together with Medical Subject Headings (MeSH) terms. Searches were also limited to include only English documents and if possible exclude review articles. The search strings contained the following search terms: ‘Africa’ AND ‘toxoplasma’ OR ‘toxoplasmosis’ AND ‘genotype’ OR ‘genetic’ OR ‘diversity’ OR ‘molecular’ OR ‘PCR’ OR ‘strain*’ OR ‘markers’ (Table S1).

### Eligibility criteria

English records reporting on *T. gondii* genotypes in African countries or genotyped isolates that originated from an African country were included. Records encompassed journal articles, preprint articles, conference papers, clinical case reports and dissertations or theses.

Exclusion criteria consisted of the following:

- publications that did not describe *T. gondii* genotypes in or from Africa;
- records where the data were from previous studies already included in this review;
- the full text was not retrievable;
- details of genotypes were not indicated;
- isolates that did not originate from African countries;
- origin of isolates not indicated;
- insufficient detail on the methods used and genotypes found, reports on research methods only;
- review articles and books or book chapters.

### Selection process

The records retrieved from the databases were exported into RefWorks reference manager. The ‘find duplicates’ functions were used to remove duplicate documents. The remaining records were then exported to an Excel spreadsheet and tabulated showing the reference ID, authors, title, periodical, publication year, abstract, notes, publisher, links and DOI of each record. Duplicates that were missed during RefWorks screening were removed by using the ‘find and highlight duplicates’ function in Excel. Screening was done for relevancy on the remaining records. Titles and abstracts were screened by 1 reviewer based on the eligibility criteria; if the information was unclear in the abstract, the full text was screened to retrieve more information. Excel spreadsheets were used throughout the process, highlighting studies either for further assessment or exclusion. Searching and screening processes were performed by 1 reviewer and were verified by 3 other reviewers.

### Data extraction and analysis

After the screening process, records highlighted for comprehensive analysis were analysed in detail by 1 reviewer and the results were verified by 3 other reviewers. Records that did not contain enough data to fulfil the eligible outcomes were not included in the review. Data outcomes encompassed year, first author, country, host species, population details, sample types, sample size, diagnostic techniques, genotyping method, molecular markers, number of isolates, genotyping results and isolate names (if stated). RFLP genotype (ToxoDB) designations were not indicated in all the records. However, some of the published isolates were used in the development of the ToxoDB database (Su *et al*., 2012; Shwab *et al*., 2014). RFLP genotype information for some of the isolates was retrieved by searching the ToxoDB database for isolates from African countries and the specific isolate names indicated in the reviewed records. Data from the results in the included studies were tabulated into Excel spreadsheets and descriptively analysed. Various functions in Excel were used to sort, combine and visualize the data into tables and charts. Distribution maps were created using the free and Open Source QGIS software version 3.26.0 (QGIS Association, 2022). Cohen's *D* effect size was calculated to determine the difference between the means of the results when including and excluding studies with less than 5 molecular markers; the formula and interpretation from Glen (2022) were used in combination with Excel tools.

## Results

### Study selection

**Fig. 1.**
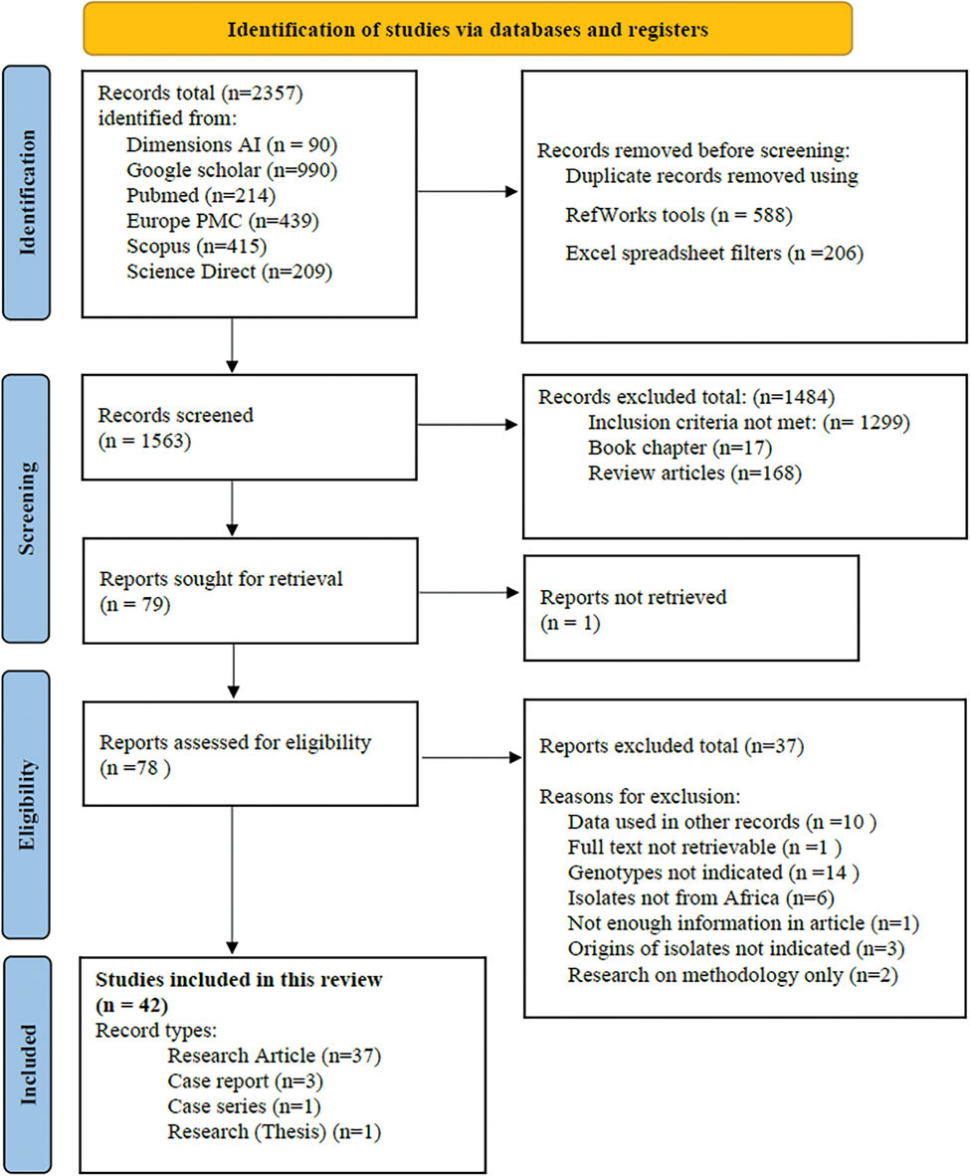
Flow diagram of the search and selection process for the publications.

The selection process is shown (Fig. 1). A total of 2357 records were retrieved from the 6 databases. The coverage dates for records retrieved from the respective databases ranged from 1915 to 2022 (Table S1). During the title/abstract screening process, 1484 records were excluded (17 book chapters, 168 review articles and 1299 other records) that did not meet the inclusion criteria. The remaining 79 records were assessed in their entirety and another 37 records were excluded. Reasons for exclusion of these articles include the full text of 1 article was not available, genotyping data were already used in other records included in the review (*n* = 10), genotypes were not indicated in the results (*n* = 14), isolates were not from African countries (*n* = 6), origins of isolates were not clearly indicated (*n* = 3), research was done on methodology only (*n* = 2) and 1 record with not enough information for assessment. Forty-two records were found appropriate for inclusion in this systematic review, these included 37 research papers, 3 case reports, 1 case series and 1 published thesis.

### Study characteristics

**Fig. 2.**
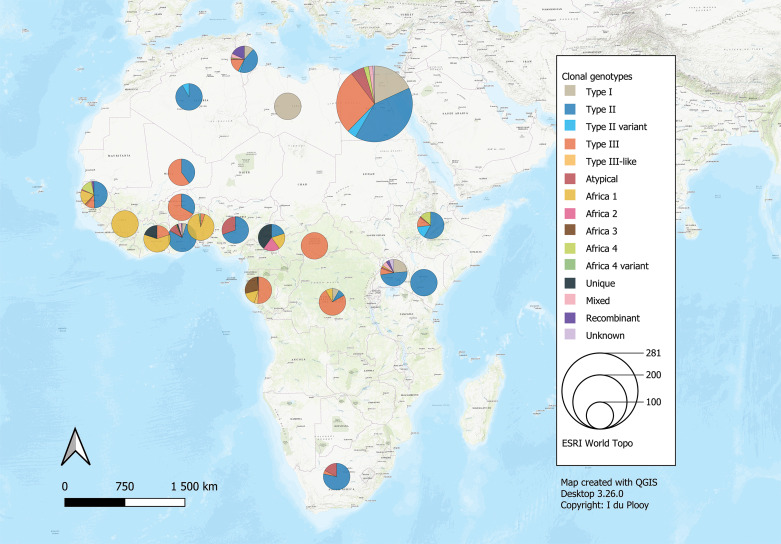
Geographical distribution of *T. gondii* genotypes in Africa, according to clonal genotype designations. The sizes of the pie charts correlate with the total number of isolates and colours are representative of the different genotypes.

**Fig. 3.**
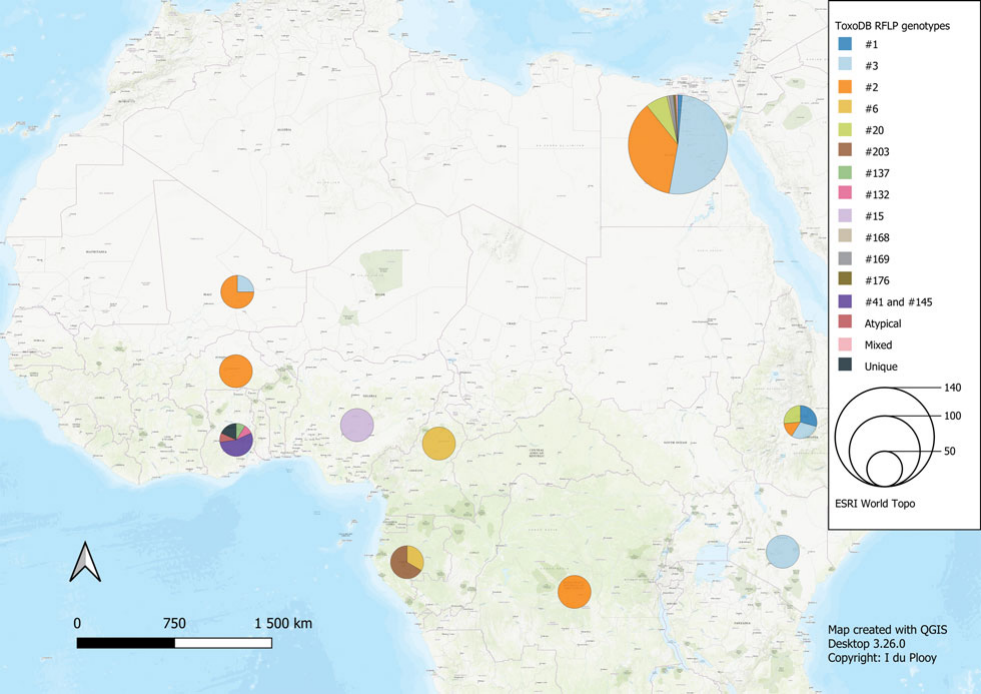
Geographical distribution of *T. gondii* genotypes in Africa, according to ToxoDB PCR-RFLP genotype designations. The sizes of the pie charts correlate with the total number of isolates and colours are representative of the different genotypes.

The 42 reviewed records consisted of 70 distinct datasets, some studies conducted genotyping on more than 1 type of host or in more than 1 country. An overview of the characteristics of the studies can be seen in Table 1. Sample data were obtained from 21 different host species, 1383 samples from humans and 9074 animal samples, making up the total sample size of 10 457. A total of 885 *T. gondii* isolates from 20 countries were characterized into archetypal clonal lineages (types I, II and III), clonal variants, regional or local clonal lineages (*Africa 1–4*), unique strains, mixed types, recombinant strains and unknown types (Table 2, Fig. 2). The PCR-RFLP ToxoDB genotypes of 203 of these isolates, originating from 10 of the countries, were identified as ToxoDB#1, #2, #3, #6, #15, #20, #41/#145, #132, #137, #168, #169, #176, #203, atypical strains, mixed types and unique types (Table 3, Fig. 3). In the 70 datasets, the genotyping methods were based on multiplex PCR of MS markers, PCR-RFLP and sequencing-only methods (Table 4). The number of molecular markers varied across the studies, ranging from 1 to 15 markers, with multilocus typing (genotyping with 5 or more markers) being used in 70% of the datasets.

**Table 1.** Summary of study characteristics for records used in this review

Reference	Country	Host species	Population details	Sample types	Diagnostic techniques	No. of molecular markers	Clonal genotype (No.)	RFLP ToxoDB genotype (No.)	Sample size
Abd El-Razik *et al*. (2018)	Egypt	Goat *(Capra aegagrus hircus)*	Goats from abattoirs in Cairo and Giza	Serum, tissue samples from diaphragm, heart and thigh muscles	OnSite Toxo IgG/IgM rapid test cassettes, ELISA, microscopy, mouse bioassay, PCR (B1), nested PCR-RFLP	2	Type II (3)	ND	51
		Sheep *(Ovis aries)*	Sheep from abattoirs in Cairo and Giza				Type II (7) Type III (6) Atypical (2)	ND	193
Ajzenberg *et al*. (2009)	Benin	Human	Immunocompromised patients	Cerebral biopsy	Cell culture, mouse tissue culture, multiplex PCR of MS markers	6	*Africa 1* (1)	ND	1
	Cameroon			Cerebral biopsy, CSF, brain			Type II (1) *Africa 1* (1) *Africa 2* (1) Unique (2)	#6 (1)^a^	5
	Central African Republic			Cerebral biopsy			Type III (1)	ND	1
	Côte d'Ivoire			Sputum, cerebral biopsy, spinal cord abscess, blood	Multiplex PCR of MS markers		Type III (1) *Africa 1* (2) Unique (1)	ND	4
	Democratic Republic of Congo			Cerebral biopsy	Cell culture, mouse tissue culture, multiplex PCR of MS markers		Type III (1)	ND	1
	Ghana			Cerebral biopsy	Multiplex PCR of MS markers		*Africa 1* (1)	ND	1
	Guinea			Myocardial biopsy	Cell culture, mouse tissue culture, multiplex PCR of MS markers		*Africa 1* (1)	ND	1
	Senegal			Blood, bronchoalveolar lavage fluid			*Africa 1* (1) *Africa 2* (1)	ND	2
	Tunisia			Cerebral biopsy			Type II (1)	ND	1
	Uganda			Cerebral biopsy	Multiplex PCR of MS markers		*Africa 1* (1)	ND	1
Ali *et al*. (2018)	Libya	Human	HIV negative, ocular toxoplasmosis patients at eye hospital in Benghazi, Libya	Serum	ELISA, nested PCR (B1), nested PCR-RFLP	2	Type I (6)	ND	43
Al-Kappany *et al*. (2010)	Egypt	Cat (*Felis catus*)	Feral cats from Abourawash, Giza, Egypt	Brain, tongue, heart	Mouse bioassay, cell culture (CV1 cells), multiplex multilocus nested PCR-RFLP	10	Type II (61) Type III (42) Atypical (8) Mixed type II/III (4)	#3 (59)^a^ #20 (4) #2 (42) #176 (1) #169 (2) #168 (1) #1 (2)	137
Ayi *et al*. (2016)	Ghana	Human	Immune compromised and immune competent individuals in Accra, Ghana	Citrated Blood	nested PCR-RFLP	3	Type I (3) Type II (79) Mixed Type I and II (4)	ND	297
Badr *et al*. (2016)	Egypt	Human	Women, 1st trimester pregnancy terminated	Serum, placenta	ELISA, qPCR (B1), nested PCR-RFLP	2	Type I (4) Type II (3) Unknown (2)	ND	72
Bamba *et al*. (2016)	Burkina Faso	Pig (*Sus domesticus*)	Pig carcasses from abattoir in Burkina Faso	Diaphragm, heart	ELISA, qPCR (targeting AF487550), SNPs in minisequencing	8 SNPs	Type II (1) Type III (1)	ND	300
Boughattas *et al*. (2010)	Tunisia	Human	Cases of congenital toxoplasmosis	Amniotic fluid, placenta, CSF	n-PCR-RFLP, sequencing	6	Type I (1) Recombinant type I/III (7) Recombinant type I/II (3) Mix recombinant I/II and I/III (3)	ND	14
Boughattas *et al*. (2011*a*)	Tunisia	Human	Pregnant female	Amniotic fluid	Real-time PCR (B1 and Rep529), Mn-PCR-RFLP, sequencing	10	Atypical (Combination of types I, II, III and non-archetypal alleles) (1)	ND	1
Boughattas *et al*. (2011*b*)	Tunisia	Human	Pregnant female and infant	Serum, placenta, amniotic fluid, fetus, cord blood, eyes	ELISA, western blot, qPCR (B1 and Rep529), mouse bioassay, ultrasonography, ophthalmologic fundi, Mn-PCR-RFLP, sequencing	7	Recombinant I/III (1)	ND	1
Boughattas *et al*. (2014)	Tunisia	Sheep (*Ovis aries*)	Ewes heart from butchers in Tunis city	Heart	Modified agglutination test, PCR (B1), nested PCR-RFLP, sequencing	1	Type I (2) Type II (2) Type III (10) Type II/III Mix (4)	ND	72
Dubey *et al*. (2003)	Egypt	Chicken (*Gallus domesticus*)	Free range chickens from rural area surrounding Giza, Egypt	Serum, heart, brain	Modified agglutination test (MAT), mouse bioassay, cat bioassay, nested PCR-RFLP	2	Type II (3) Type III (17)	#2 (5)^a^ #3 (2)	121
		Duck (*Anas species*)	Ducks from rural area surrounding Giza, Egypt				Type III (1)	ND	19
Dubey *et al*. (2005)	Burkina Faso	Chicken (*Gallus domesticus*)	Chickens purchased at local market	Brain, heart	Mouse bioassay, nested PCR-RFLP	2	Type III (1)	#2 (1)^a^	40
	Democratic Republic of Congo		Chickens from households near Kinshasa, DRC	Serum, heart, pectoral muscle, brain	Modified agglutination test (MAT), mouse bioassay, cat bioassay, nested PCR-RFLP		Type I (1) Type II (1) Type III (8)	#2 (4)^a^	50
	Kenya		Chickens from slaughterhouse in Kisumu, Kenya	Serum, brain			Type II (1)	#3 (1)^a^	30
	Mali		Chickens purchased at local market	Brain, heart	Mouse bioassay, nested PCR-RFLP		Type II (2) Type III (3)	#2 (3)^a^ #3 (1)	48
Dubey *et al*. (2008)	Ghana	Chicken (*Gallus domesticus*)	Chickens purchased from the market for free-range chickens in Kumasi, Ghana	Brain, whole heart and blood	Modified agglutination test (MAT), mouse and cat bioassays, Mn-PCR-RFLP	11	Atypical (2)	#137^a^ #132	64
Dubey *et al*. (2013)	Ethiopia	Cat (*Felis catus*)	Feral cats from Addis Ababa, Ethiopia	Heart, feces	Mouse bioassay, cell culture, multiplex multilocus nested PCR-RFLP	10	Type II (ToxoDB#1) (9) Type III (ToxoDB#2) (5) Type II variant (ToxoDB#3) (10) *Africa 4* (ToxoDB#20) (9)	#1 (9) #2 (5) #3 (10) #20 (9)	27
El Behairy *et al*. (2013)	Egypt	Dog (*Canis familiaris*)	Stray dogs captured from Abourawash, Giza, Egypt	Blood, serum, heart	Modified agglutination test (MAT); mouse bioassay; *in vitro* cell culture; Mn-PCR-RFLP	11	Type II-variant (ToxoDB#3) (11) *Africa 4* (ToxoDB#20) (6) Type III (ToxoDB#2) (4) Mixed (1)	#3 (11) #20 (6) #2 (4) Mixed (1)	51
El Fadaly *et al*. (2016)	Egypt	Rat (*Rattus* species)	Different species of rats from different rural and urban sites in Cairo and Giza, Egypt	Serum, brain, liver, kidney, heart	Latex agglutination test, ELISA, mouse bioassay, kitten bioassay, nested PCR-RFLP, histopathology stains and microscopy	2	Type I (3) Type II (9) Type III (5)	ND	278
El-Alfy *et al*. (2019)	Egypt	One-humped camel (*Camelus dromedarius*)	Slaughtered one-humped camels at Al Bassatein abattoir, Cairo, Egypt	Heart	PCR (B1), Mn-PCR-RFLP, sequencing	12	Type II (1)	ND	90
Eldeek *et al*. (2017)	Egypt	Human	Pregnant women with adverse pregnancy outcomes	Placental and/or trophoblastic tissues	ELISA, PCR (B1), multiplex nested PCR-RFLP	5	Type I (28) Atypical (1)	ND	29
Gad *et al*. (2022)	Egypt	Sheep (*Ovis aries*)	Samples from live ewes and slaughtered ewes from governorates in Egypt	Serum, milk, heart, diaphragm, uterus, thigh muscles	ELISA, mouse bioassay, histopathology microscopy, PCR (B1), nested PCR-RFLP	2	Type II (14) Type III (2)	ND	318
		Goat (*Capra aegagrus hircus*)	Samples from live she-goats and slaughtered she-goats from governorates in Egypt				Type II (12)	ND	418
Galal *et al*. (2019*b*)	Senegal	Poultry, domestic and wild animals (chicken *Gallus domesticus*, Muscovy duck *Cairina moschata*, Mallard duck *Anas platyrhynchos*, guinea fowl *Numida meleagris*, cat *Felis catus*, sheep *Ovis aries*, francolin *Pternistis bicalcaratus*)	Poultry raised around households and occasionally opportunistic sampling of other domestic or wild animals when they were available	Serum, brain, heart	Modified agglutination test, mouse bioassay, multiplex PCR of MS markers, sequencing	15	Type II (37) Type III (7) *Africa 1* (13) *Africa 4* (13) Recombinant III/*Africa 1* (2)	ND	2040
Galal *et al*. (2019*c*)	Senegal	Rodents and shrews (giant pouch rat *Cricetomys gambianus*, shrew *Crocidura olivieri*, house mouse *Mus musculus domesticus*)	Rodents and shrews trapped in districts of Senegal	Serum, brain	Modified agglutination test, qPCR (REP529), multiplex PCR of MS markers	15	Type II (6) Type III (2) Type III-like (1) *Africa 1* (2) Atypical (1)	ND	828
Gebremedhin *et al*. (2014)	Ethiopia	Goat (*Capra aegagrus hircus*)	Slaughtered goats from abattoir	Serum, heart	Direct agglutination test, mouse bioassay, multiplex PCR of MS markers, sequencing	15	Type II (13) Type III (2)	ND	44
		Sheep (*Ovis aries*)	Slaughtered sheep from abattoir				Type II (16) Type III (1) Atypical (1)	ND	47
Genot *et al*. (2007)	Ghana	Human	African HIV-positive patient	Brain biopsy, CSF	MRI, real-time PCR (Rep529), histopathology, multiplex PCR of MS markers	5	Recombinant I/III	ND	1
Ghozzi *et al*. (2017)	Tunisia	Marine bivalve molluscs	Marine bivalve molluscs collected along Tunisian coasts, positives from *Ruditapes decussatus* (grooved carpet shell clam)	Mollusc flesh	qPCR (B1), sequencing	na	Type I (6)	ND	1255
Hamidović *et al*. (2021)	Benin	Poultry (chicken *Gallus domesticus*, Mallard duck *Anas platyrhynchos*)	Hens, ducks, guinea fowl, turkey from 4 cities of Benin	Serum, heart, brain	Modified agglutination test, mouse bioassay, multiplex PCR of MS markers	15	*Africa 1* (36) Type III (2) *Africa 4*-variant (1)	ND	758
Hammond-Aryee (2016)	South Africa	Cat (*Felis catus*)	Post-mortem examinations of *T. gondii*-infected kittens	Lung, intestine	PCR (B1), multiplex PCR of MS markers, sequencing	15	Type II (1) Atypical (2)	ND	3
		Human	Male and female patients presenting with acute *T. gondii* infection	Whole blood, eye fluid and cerebrospinal fluid		8	Type II (8) Type III (1) Atypical (2)	ND	17
		Marmoset (*Callithrix* spp)	Post-mortem examinations of *T. gondii*-infected marmoset	Intestine, liver		15	Type II (2)	ND	2
		Squirrel monkey (*Saimiri* spp)	Post-mortem examinations of *T. gondii*-infected squirrel monkeys	Tissues, brain, liver, lung, intestines			Type II (6)	ND	6
Lachkhem *et al*. (2021)	Tunisia	Chicken (*Gallus domesticus*)	Free-range chickens from farms/backyards	Serum, heart, brain	Direct agglutination test, mouse bioassay, PCR (REP529), multiplex PCR of MS markers	15	Type II (10) Type II-variant (2)	ND	136
		Sheep (*Ovis aries*)	Sheep from slaughterhouses	Serum, heart			Type II (17) Type III (3) *Africa 4* (1)	ND	630
Lahmar *et al*. (2020)	Tunisia	Human	Pregnant women with Toxoplasmosis	Amniotic fluid, placenta	PCR (REP529), mouse bioassay, indirect fluorescent antibody test, multiplex PCR of MS markers	15	Type II (4)	ND	80
Leroy *et al*. (2020)	Côte d'Ivoire	Human	Immunocompetent individuals with acute acquired toxoplasmosis	Serum, blood, bronchoalveolar lavage, bone marrow	ELISA, PCR, multiplex PCR of MS markers	15	*Africa 1* (1)	ND	1
	Democratic Republic of Congo	Human		Serum, axillary lymph node biopsy			*Africa 1* (1)	ND	1
	Senegal	Human		Serum, CSF, aqueous humour, muscle biopsy			Africa or recombinant I/III (1)	ND	2
Lindström *et al*. (2006)	Uganda	Human	HIV-positive males and females	Blood, serum, buffy-coat	Direct agglutination test, PCR (B1), nested PCR-RFLP	3	Type I (6) Type II (17) Type III (3) Recombinant I/III (1) Unknown (3)	ND	130
Lindström *et al*. (2008) and Bontell *et al*. (2009)	Uganda	Chicken (*Gallus domesticus*)	Free range chickens from households in and around Kampala, Uganda	Serum, heart, brain	Modified agglutination test (MAT), mouse and cat bioassays, Mn-PCR-RFLP, sequencing	5	Type I (6) Type II (8) Type III (1) Atypical (1) Recombinant II/III (1) Mix type I/II (3)	ND	85
Lukášová *et al*. (2018)	South Africa	Red eyed dove (*Streptopelia semitorquata*)	Wild and domestic birds from Limpopo, South Africa	Brain	PCR (B1), multiplex PCR of MS markers	15	Type II (1)	ND	110
Mercier *et al*. (2010)	Gabon	Goat (*Capra aegagrus hircus*)	Goats from Dienga, Gabon	Serum, brain, heart	Modified agglutination tests, real-time quantitative PCR (REP529), mouse bioassay, multiplex PCR of MS markers, sequencing	13	Type III (8) Type III-like (2)	ND	12
		Sheep (*Ovis aries*)	Sheep from Dienga, Gabon				Type III (7)	ND	7
		Cat (*Felis catus*)	Domestic cat from Dienga, Gabon				Type III (1)	ND	1
		Chicken (*Gallus domesticus*)	Free-range chickens from localities in Gabon				Type III (19) Type III-like (1) *Africa 1* (11) *Africa 3* (19) Single genotype (1)	#203 (4)^a^ #6 (2)	53
Nassef *et al*. (2015)	Egypt	Human	Pregnant females	Serum, placenta and POC	ELISA, PCR (B1), nested PCR-RFLP	2	Type I (9)	ND	92
Nzelu *et al*. (2021)	Nigeria	Chicken (*Gallus domesticus*)	Free-range chickens from live bird markets	Thigh muscle	Nested PCR (529REP), quantitative PCR, nested PCR-RFLP, sequencing, MLST	5	Type II (6) Atypical (4)	ND	173
		Human	*Toxoplasma gondii* seropositive pregnant women	Serum, whole blood on Whatman FTA cards	ELISA, nested PCR (529REP), quantitative PCR, nested PCR-RFLP, sequencing, MLST		Type II (6) Atypical (4)	ND	91
		Pig (*Sus domesticus*)	Slaughter slabs for pigs	Thigh muscle	Nested PCR (529REP), quantitative PCR, nested PCR-RFLP, sequencing, MLST		Type II (11) Atypical (1)	ND	211
Pappoe *et al*. (2017)	Ghana	Chicken (*Gallus domesticus*)	Free-range chickens from restaurant and market	Brain	Nested PCR (gra6), n-PCR-RFLP, sequencing	10	Atypical (ToxoDB#41 and #145) (5) Atypical (identical to TgCkBrRj2) (1)	#41 and #145 (5) Atypical (identical to TgCkBrRj2) (1)	25
		Human	HIV-infected individuals	Blood, plasma, buffy coat	ELISA, nested PCR (gra6), n-PCR-RFLP, sequencing		Partial typing at SAG3 only: Type I (2) Type II (3) Type III (1)	ND	394
		Cat (*Felis catus*)	Cats slaughtered for human consumption from restaurant	Brain	Nested PCR (gra6), n-PCR-RFLP, sequencing		Atypical (ToxoDB#41 and #145) (1) Atypical (new unique TgCtGh1) (2)	#41 and #145 (1) Atypical (new unique TgCtGh1) (2)	40
Tilahun *et al*. (2013)	Ethiopia	Chicken (*Gallus domesticus*)	Backyard local species of chickens from the 10 districts of Addis Ababa, Ethiopia	Serum, heart	Modified agglutination test, mouse bioassay, cat bioassay, cell culture (CV1), Mn-PCR-RFLP	12	Type II (ToxoDB#1) (1)	#1 (1)	125
Tolba *et al*. (2014)	Egypt	Human	Individuals with detectable anti-toxoplasma antibodies in serum samples	Blood	Nested PCR-RFLP	1	Type I (7) Atypical (5)	ND	100
Velmurugan *et al*. (2008)	Nigeria	Chicken (*Gallus domesticus*)	Chickens were purchased from a market in Vom, Plateau, Nigeria	Serum, heart	Modified agglutination test (MAT), mouse bioassay, Mn-PCR-RFLP	10	Atypical (1)	#15 (1)^a^	79
Yekkour *et al*. (2017)	Algeria	Cat (*Felis catus*)	Stray cats in Algiers, Algeria	Blood, heart, spleen, tongue	Modified agglutination test, qPCR (REP529), multiplex PCR of MS markers	15	Type II (11) Type II-variant (1)	ND	96
Total: 42 records, 70 datasets	**20**	**21**					**885**	**203**	**10 457**

MS, microsatellite marker; ND, not determined; Mn-PCR-RFLP, multilocus nested-PCR-RFLP.

a Genotype on ToxoDB database.

**Table 2.** Clonal genotypes of *T. gondii* from reviewed studies, grouped according to country

Country	Type I	Type II	Type II-var	Type III		Type III-like	*Africa 1*	*Africa 2*	*Africa 3*	*Africa 4*	*Africa 4*-var	Atyp	Unk	Mix	Recomb	Unq	No. of isolates per country (%)
Algeria		11	1														12 (1.3)
Benin					2		37				1						40 (4.52)
Burkina Faso		1			2												3 (0.34)
Cameroon		1					1	1					2				5 (0.56)
Central African Republic					1												1 (0.11)
Côte d'Ivoire					1		3						1				5 (0.56)
Democratic Republic of Congo	1	1			9		1										12 (1.36)
Egypt	51	113	11		77					6		16		5		2	281 (31.75)
Ethiopia		39	10		8					9		1					67 (7.57)
Gabon					35	3	11		19				1				69 (7.8)
Ghana	5	82			1		1					9	2	4	1		105 (11.86)
Guinea							1										1 (0.11)
Kenya		1															1 (0.11)
Libya	6																6 (0.68)
Mali		2			3												5 (0.56)
Nigeria		23										10					33 (3.73)
Senegal		43			9	1	16	1		13		1		1	2		87 (9.83)
South Africa		18			1							4					23 (2.60)
Tunisia	9	34	2		13					1		1		4	14		78 (8.81)
Uganda	12	25			4		1					1			2	6	51 (5.76)
No. of isolates per genotype (%)	84 (9.49)	394 (44.52)	24 (2.71)		166 (18.76)	4 (0.45)	72 (8.14)	2 (0.23)	19 (2.15)	29 (3.28)	1 (0.11)	43 (4.86)	6 (0.68)	14 (1.92)	19 (2.15)	8 (0.56)	885 (100)

var, variant; Atyp, atypical; Unk, unknown; Mix, mixed; Recom, recombinant; Unq, unique.

**Table 3.** ToxoDB PCR-RFLP genotypes of *T. gondii* from reviewed studies, grouped according to country

Country	ToxoDB number																No. of ToxoDB isolates per country (%)
	#132	#1	#137	#15	#168	#169	#176	#2	#20	#203	#3	#41 and #145	#6	Atyp	Mix	Unq	
Burkina Faso								1									1 (0.49)
Cameroon													1				1 (0.49)
Democratic Republic of Congo								4									4 (1.97)
Egypt		2			1	2	1	51	10		72				1		140 (68.97)
Ethiopia		10						5	9		10						34 (16.75)
Gabon										4			2				6 (2.96)
Ghana	1		1									6		1		2	11 (5.42)
Kenya											1						1 (0.49)
Mali								3			1						4 (1.97)
Nigeria				1													1 (0.49)
No. of isolates per ToxoDB genotype (%)	1 (0.5)	12 (5.9)	1 (0.5)	1 (0.5)	1 (0.5)	2 (1)	1 (0.5)	64 (31.5)	19 (9.4)	4 (2)	84 (41.4)	6 (3)	3 (1.5)	1 (0.5)	1 (0.5)	2 (1)	203 (100)

Atyp, atypical; Mix, mixed; Unq, unique.

**Table 4.** Molecular markers used in the reviewed studies

Genotyping method	No. of molecular markers	Molecular markers	Reference	PCR-RFLP-based datasets	MS-based datasets	Sequencing-based datasets
High-resolution melting and mini-sequencing analyses of the repeated B1 gene	8 SNPs	NA	Bamba *et al*. (2016)			1
qPCR (B1), sequencing	NA	NA	Ghozzi *et al*. (2017)			1
Mn-PCR-RFLP	5	3′ *SAG2*, 5′ *SAG2*, *SAG3*, *GRA6*, *BTUB*	Eldeek *et al*. (2017)	1		
	10	*SAG1*, *SAG2*, *SAG3*, *BTUB*, *GRA6*, *c22-8*, *c29-2*, *L358*, *PK1*, *Apico*	Velmurugan *et al*. (2008)	1		
			Al-Kappany *et al*. (2010)	1		
			Dubey *et al*. (2013)	1		
	11	*SAG1*, *SAG2*, New *SAG2*, *SAG3*, *BTUB*, *GRA6*, *c22-8*, *c29-2*, *L358*, *PK1*, *Apico*	Dubey *et al*. (2008)	1		
			El Behairy *et al*. (2013)	1		
	12	*SAG1*, *SAG2* (5′-*SAG2* and 3′-*SAG2*), alt. *SAG2*, *SAG3*, *BTUB*, *GRA6*, *c22-8*, *c29-2*, *L358*, *PK1*, *Apico*	Tilahun *et al*. (2013)	1		
			El-Alfy *et al*. (2019) (successful for *SAG2* and alt. *SAG2* only)	1		
Mn-PCR-RFLP, sequencing	5	*SAG1*, *SAG2*, *SAG3*, *BTUB*, *GRA6*	Lindström *et al*. (2008) and Bontell *et al*. (2009)	1		
	7	3′*SAG2*, 5′*SAG2*, *SAG3*, *Apico*, *AK69*, *UPRT1*, *DHPS*	Boughattas *et al*. (2011*b*)	1		
	10	3′*SAG2*, 5′*SAG2*, new *SAG2*, *SAG3*, *BTUB*, *GRA6*, *PK1*, KT-850, *Apico*, *UPRT1*	Boughattas *et al*. (2011*a*)	1		
Multiplex PCR of MS markers	5	*TUB2*, *TgM-A*, *W35*, *B17*, *B18*	Genot *et al*. (2007)		1	
	6	*TUB2*, *W35*, *TgM-A*, *B18*, *B17*, *M33*	Ajzenberg *et al*. (2009)		10	
	13	*TUB2*, *W35*, *TgM-A*, *B18*, *B17*, *M33*, *M48*, *AA*, *N82*, *N83*, *N60*, *N61*, *M102*	Mercier *et al*. (2010)		4	
	15	*B18*, *M33*, *TUB2*, *XI.1*, *TgM-A*, *W35*, *IV.1*, *B17*, *M48*, *M102*, *N60*, *N82*, *AA*, *N61*, *N83*	Galal *et al*. (2019*c*)		1	
			Lachkhem *et al*. (2021)		2	
			Lahmar *et al*. (2020)		1	
			Leroy *et al*. (2020)		3	
			Lukášová *et al*. (2018)		1	
			Hamidović *et al*. (2021)		1	
			Yekkour *et al*. (2017)		1	
Multiplex PCR of MS markers, sequencing	8	*TUB 2*, *TgM-A*, *W35*, *N60*, *N82*, *N83*, *N61*, *AA*	Hammond-Aryee (2016)		1	
	15	*B18*, *M33*, *TUB2*, *XI.1*, *TgM-A*, *W35*, *IV.1*, *B17*, *M48*, *M102*, *N60*, *N82*, *AA*, *N61*, *N83*	Gebremedhin *et al*. (2014)		1	
			Hammond-Aryee (2016)		3	
			Gebremedhin *et al*. (2014)		1	
			Galal *et al*. (2019*b*)		1	
Nested PCR-RFLP, sequencing (*SAG3*)	1	*SAG3*	Boughattas *et al*. (2014)	1		
Nested PCR-RFLP	1	*GRA6*	Tolba *et al*. (2014)	1		
	2	3′*SAG2*, 5′*SAG2*	Abd El-Razik *et al*. (2018)	2		
			Ali *et al*. (2018)	1		
			Badr *et al*. (2016)	1		
			Dubey *et al*. (2003)	2		
			Dubey *et al*. (2005)	4		
			El Fadaly *et al*. (2016)	1		
			Gad *et al*. (2022)	2		
			Nassef *et al*. (2015)	1		
	3	3′*SAG2*, 5′*SAG2*, *BTUB* (1 isolate)	Lindström *et al*. (2006)	1		
		*SAG3*, *GRA6*, *BTUB*	Ayi *et al*. (2016)	1		
Nested PCR-RFLP, sequencing, MLST	5	*SAG3*, *SAG2* (5′ and 3′), *BTUB*, *GRA6*, *Apico*	Nzelu *et al*. (2021)	3		
n-PCR-RFLP, sequencing	6	3′*SAG2*, 5′ *SAG2*, *SAG3*, *BTUB*, *GRA6*, *Apico*	Boughattas *et al*. (2010)	1		
	10	*SAG 1*, Alt. SAG 2, SAG 3, *BTUB*, *GRA6*, *C22-8*, *C29-2*, *L358*, *PK1*, *Apico*	Pappoe *et al*. (2017)	3		
Total				36	32	2
Percentage				51%	46%	3%

NA, not applicable; SNPs, single-nucleotide polymorphisms.

### Genetic diversity in Africa

#### Northern Africa

Studies from Northern Africa (Egypt, Tunisia, Algeria, Libya) made up 35.7% (25/70) of the total datasets in this review. A total of 377 *T. gondii* isolates were recovered from 10 different host species with most isolates recovered from cats (127 isolates) and the least isolated recovered from ducks (1 isolate) and a one-humped camel (1 isolate) (Table 1). Records from Egypt were the most out of all the countries, with 31.75% of clonal type isolates (281/885) as well as 68.97% (140/203) of ToxoDB categorized isolates (Tables 2 and 3). The most prevalent clonal genotypes in Egyptian studies were type II (113/281) followed by type III (77/281) and type I (51/281) (Table 2). Other clonal types consisted of atypical (16/281), type II variants (11/281), 6 *Africa 4* types, 5 mixed types and 2 unknown types. ToxoDB#3 (72/140) was the most dominant of the PCR-RFLP defined types, followed by ToxoDB#2 (51/140) and ToxoDB#20 (10/140). Isolated cases of ToxoDB#1, #168, #169, #176 and a mixed type were also found. ToxoDB isolate IDs have been assigned to 118/140 ToxoDB RFLP genotyped isolates from cats, dogs and chickens in Egypt (Table 5). Atypical genotypes, unique to Egypt included ToxoDB#168, #169 and #176 (Table 6). Tunisia was like Egypt, in that clonal types were dominated by type II (34/78) genotypes but instead followed by recombinant genotypes (types I/III and I/II, 14/78), type III (13/78) and type I (9/78) (Tables 1 and 2). Clonal type II variant, *Africa 4* and mixed genotypes were present in low amounts. Only 6 type I clonal isolates were identified in Libya, samples were from ocular toxoplasmosis patients at an eye hospital in Benghazi (Ali *et al*., 2018). In Algeria, only clonal type II (11) genotypes and 1 type II variant was found in samples of stray cats in Algiers. No ToxoDB genotypes were identified from Tunisia, Algeria or Libya.

**Table 5.** Details of *T. gondii* isolates characterized according to PCR-RFLP ToxoDB genotypes

Reference	Country	Species	Clonal genotype	RFLP ToxoDB genotype	ToxoDB isolate ID	Isolate description (strain ID)	No. of ToxoDB isolates
Ajzenberg *et al*. (2009)	Cameroon	Human	*Africa 1*	#6	EUSMPL0040-1-140	PSP-2003-KOM (PSP-KOM)	1
Al-Kappany *et al*. (2010)	Egypt	Cat	Atypical	#168	EUSMPL0040-1-108	TgCatEg57	1
				#169	EUSMPL0040-1-109 to 110	TgCatEg53,67	2
				#20	EUSMPL0040-1-104 to 107	TgCatEg7,18,34,65	4
			Atypical	#176	EUSMPL0040-1-111	TgCatEg88	1
			Type II	#1	EUSMPL0040-1-1 to 2	TgCatEg115, 55	2
			Type III	#2	EUSMPL0040-1-3 to EUSMPL0040-1-44	TgCatEg6, 11, 13, 15, 17, 19, 22, 24, 35, 36, 37, 43, 47, 48, 50, 58, 61, 62, 66, 69, 70, 71, 72, 73, 74, 80, 82, 83, 84, 86, 87, 92, 93, 94, 95, 100, 102, 105, 108, 110, 111, 114	42
			Type II-variant	#3	EUSMPL0040-1-45 to EUSMPL0040-1-103	TgCatEg 1, 2, 3, 4, 5, 8, 9, 10, 12, 14, 16, 20, 21, 23, 25, 26, 28, 29, 30, 31, 32, 33, 38, 39, 41, 42, 44, 45, 46, 49, 51, 52, 54, 56, 59, 60, 63, 64, 68, 75, 76, 77, 78, 79, 81, 85, 90, 91, 96, 97, 98, 99, 101, 103, 104, 107, 109, 112, 113	59
Dubey *et al*. (2003)	Egypt	Chicken	Type III	#2	EUSMPL0040-1-117 to 121	TgCkEg12,13,14,16,17	5
			Type II-variant	#3	EUSMPL0040-1-125 to 126	TgCkEg15,19	2
Dubey *et al*. (2005)	Burkina Faso	Chicken	Type III	#2	EUSMPL0040-1-112	TgCkBF-1	1
	Democratic Republic of Congo	Chicken	Type III	#2	EUSMPL0040-1-113 to 116	TgCkDROC-3,6,8,9	4
	Kenya	Chicken	Type II-variant	#3	EUSMPL0040-1-127	TgCkKen-1	1
	Mali	Chicken	Type III	#2	EUSMPL0040-1-122 to 124	TgCkMal-2, 3, 4	3
			Type II-variant	#3	EUSMPL0040-1-128	TgCkMal-5	1
Dubey *et al*. (2008)	Ghana	Chicken	Atypical	#132	EUSMPL0040-1-132	TgCkGh2	1
				#137	EUSMPL0040-1-133	TgCkGh1	1
Dubey *et al*. (2013)	Ethiopia	Cat	*Africa 4*	#20	Not allocated	TgCatEt 11a, 11b 15a, 18a, 20a, 22a, 23a, 25a, 27a	9
			Type II	#1	Not allocated	TgCatEt 2b, 3b, 6a, 7a, 7b, 13a, 13b, 24a, 26a	9
			Type III	#2	Not allocated	TgCatEt 4a, 6b, 9b, 10a, 14a	5
			Type II-variant	#3	Not allocated	TgCatEt 1a, 3a, 5a, 9a, 12a, 16a, 17a, 19a, 21a, 28a	10
El Behairy *et al*. (2013)	Egypt	Dog	Mixed	Mixed	Not allocated	TgDogEg7	1
			*Africa 4*	#20	Not allocated	TgDogEg1,2,3,6,10,22	6
			Type III	#2	Not allocated	TgDogEg5, 13, 15, 17	4
			Type II-variant	#3	Not allocated	TgDogEg4,8, 9, 11, 12, 14, 16, 18, 19, 20, 21	11
Mercier *et al*. (2010)	Gabon	Chicken	*Africa 3*	#203	EUSMPL0040-1-134 to 137	P24, PF26, PL15, PM2 (strain ID from Shwab *et al*., 2014)	4
			*Africa 1*	#6	EUSMPL0040-1-129 to 130	PL8, S1PF2 (strain ID from Shwab *et al*., 2014)	2
Pappoe *et al*. (2017)	Ghana	Cat	Unique	Unique	Not allocated	TgCtGh1	2
		Chicken	Atypical	#41 and #145	Not allocated	TgCkCC1, 2, 3, 4, 5, 6	5
				Atypical	Identical to: TgCkBrRj2 = EUSMPL0041-1-1	Identical to: TgCkBrRj2	1
Pappoe *et al*. (2017)	Ghana	Cat	Atypical	#41 and #145	Not allocated	TgCtCC1, 2, 3	1
Tilahun *et al*. (2013)	Ethiopia	Chicken	Type II	#1	Not allocated	TgCKEt1	1
Velmurugan *et al*. (2008)	Nigeria	Chicken	Atypical	#15	EUSMPL0040-1-131	TgCkNg1	1
Total							203

**Table 6.** ToxoDB genotypes in this review shared with other geographical regions

ToxoDB RFLP genotype	Geographical regions	References
#1	Africa, Asia, Europe, North America, Central/South America	(Shwab *et al*., 2014. This review: Al-Kappany *et al*., 2010; Dubey *et al*., 2013; Tilahun *et al*., 2013)
#2	Africa, Asia, Europe, North America, Central/South America	(Shwab *et al*., 2014. This review: Dubey *et al*., 2003; Dubey *et al*., 2005; Al-Kappany *et al*., 2010; Dubey *et al*., 2013; El Behairy *et al*., 2013)
#3	Africa, Asia, Europe, North America, Central/South America	(Shwab *et al*., 2014. This review: Dubey *et al*., 2003; Dubey *et al*., 2005; Al-Kappany *et al*., 2010; Dubey *et al*., 2013; El Behairy *et al*., 2013)
#6	Africa, Asia, Europe, Central/South America	(Shwab *et al*., 2014. This review: Ajzenberg *et al*., 2009; Mercier *et al*., 2010)
#20	Africa, Asia	(Shwab *et al*., 2014. This review: Al-Kappany *et al*., 2010; Dubey *et al*., 2013; El Behairy *et al*., 2013)
#15	Africa, Europe, North America, Central/South America	(Shwab *et al*., 2014. This review: Velmurugan *et al*., 2008)
#41 and #145	Africa, Central/South America	(ToxoDB.org. This review: Pappoe *et al*., 2017)
#137	Africa (Ghana), Europe (Serbia, Bulgaria)	(ToxoDB.org. This review: Dubey *et al*., 2008; Uzelac *et al*., 2021)
#132	Africa (Ghana)	(ToxoDB.org. This review: Dubey *et al*., 2008)
#168	Africa (Egypt)	(ToxoDB.org. This review: Al-Kappany *et al*., 2010)
#169	Africa (Egypt)	(ToxoDB.org. This review: Al-Kappany *et al*., 2010)
#176	Africa (Egypt)	(ToxoDB.org. This review: Al-Kappany *et al*., 2010)
#203	Africa (Gabon)	(ToxoDB.org. This review: Mercier *et al*., 2010)

#### Eastern Africa

Studies from East African countries (Ethiopia, Kenya and Uganda) comprised 11.4% (8/70) of the total number of datasets. *Toxoplasma gondii* genotypes of 119 isolates were determined in samples sourced from chickens (22 isolates), cats (33 isolates), goats (15 isolates), sheep (18 isolates) and 31 isolates from human samples. Genotypes from Ethiopia made up 7.5% of the overall dataset. Genotypes consisted mainly of clonal type II (39/67), other genotypes found were type II variant (10/67), type III (8/67), *Africa 4* (9/67) and 1 atypical isolate. Thirty-four ToxoDB genotypes were described; they consisted of 10 isolates each of ToxoDB#1 and #3, 9 ToxoDB#20 isolates and 5 ToxoDB#2 isolates. None of these isolates has been allocated a ToxoDB isolate ID yet. Only 1 isolate was characterized from Kenya, by Dubey *et al*. (2005), the strain was isolated from a chicken and genotyped as type II with nested-PCR RFLP using 2 molecular markers. The isolate has also been characterized as ToxoDB#3, as the strain identified from the publication with the corresponding ToxoDB ID number was found on ToxoDB.org (Table 5). Sampling from Uganda revealed the presence of 51 *T. gondii* isolates which consisted of the following clonal genotypes: type II (25/51), type I (12/51), type III (4/51), 1 *Africa 1* isolate, 1 atypical isolate, 2 recombinant types (I/III and II/III), 3 mixed genotype (I/II) isolates and 3 unknown genotypes. No ToxoDB number designation could be found for these isolates.

#### Western Africa

Studies from West African countries (Benin, Burkina Faso, Côte d'Ivoire, Ghana, Guinea, Mali, Nigeria and Senegal) made up 32.9% (23/70) of the total datasets. Genotypes were determined in a total of 279 isolates from 12 different host species: chickens (25 isolates), Muscovy ducks (10 isolates), pigs (14 isolates), humans (114 isolates), Giant pouch rats (5 isolates), house mouse (6 isolates), cats (3 isolates), 2 isolates from Mallard ducks, 1 isolate each from sheep, Guinea fowl, Francolin and shrew. Eighty-seven isolates from Senegal were genotyped which constituted 9.83% of the overall dataset. Clonal type II (43/87) was dominant, other clonal types identified were *Africa 1* (16/87), *Africa 4* (13/87), type III (9/87), 2 recombinant types (III/*Africa 1*), 1 isolate each of type III-like, atypical, *Africa 2* and mixed (Africa/recombinant I/III). No ToxoDB genotypes were identified. Clonal genotypes from Ghana were the second most prevalent (11.86%) out of all the countries in this review (Table 2). Clonal types were predominantly type II (82/105), 9 atypical genotypes were described, 5 type I isolates, 4 type I/II mixed types, 2 unique genotypes, 1 recombinant type, 1 *Africa 1* isolate and 1 type III isolate. Eleven ToxoDB type isolates were isolated from chickens and cats: 1 ToxoDB#132, 1 ToxoDB#137, a mix of ToxoDB#41 and #145 (6 isolates), 1 atypical isolate identical to a Brazilian strain (TgCkBrRj2, EUSMPL0041-1-1) and 2 isolates of new unique genotype that had not been reported previously (TgCtGh1) (Tables 3 and 5). ToxoDB IDs have been assigned to 3/11 ToxoDB RFLP genotyped isolates from only chickens in Ghana (Table 5). Atypical genotypes unique to Ghana included ToxoDB#132 and #137 (Table 6). Studies from Burkina Faso yielded 3 clonal-type isolates (1 type II and 2 type III) and 1 ToxoDB#3 strain. ToxoDB isolate ID (EUSMPL0040-1-112) was assigned to this strain (Table 5). Five clonal type (2 type II, 3 type III) and 4 ToxoDB (3 #2, 1 #3) isolates were identified from chickens in Mali. ToxoDB isolate IDs were assigned to these 4 isolates (Table 5). In Nigeria, studies were done on humans, chickens and pigs. Clonal genotypes consisted of type II (23) and atypical (10) isolates, one of the atypical isolates (isolated from a chicken) was identified as ToxoDB#15 (ToxoDB ID, EUSMPL0040-1-131). The overall results of the 2 studies from Benin (40 isolates) revealed a clear dominance of the *Africa 1* genotype (37 isolates), in addition, 2 type III isolates and 1 *Africa 4* variant were identified. *Africa 1* isolates were also predominant in Côte d'Ivoire; however, only 3 isolates were identified as such. Other strains included 1 type III and 1 unique genotype. All the *T. gondii* isolates from Côte d'Ivoire were from human hosts. Only 1 *T. gondii* isolate originated from Guinea, genotyped as *Africa 1* by Ajzenberg *et al*. (2009) and isolated from a myocardial biopsy of an immunocompromised patient. No ToxoDB genotypes were identified from Benin, Côte d'Ivoire or Guinea.

#### Central Africa

Studies from Central Africa (Cameroon, Democratic Republic of Congo, Central African Republic, Gabon) encompassed 12.9% (9/70) of the total datasets. From Gabon, 1 study was found (Mercier *et al*., 2010) that genotyped a total of 69 *T. gondii* isolates from goats, sheep, chickens and cats. There was a predominance of type III (35) followed by *Africa 3* (19), *Africa 1*, type III-like and 1 unique isolate. Four of the *Africa 3* isolates were also characterized as ToxoDB#203 and 2 *Africa 1* isolates were also identified as ToxoDB#6 during the development of the ToxoDB database. Gabon is the only country where *Africa 3* and ToxoDB#203 genotypes were identified. ToxoDB IDs for these isolates are listed in Table 5. Five *T. gondii* isolates originating from samples of immunocompromised patients from Cameroon were genotyped in the study by Ajzenberg *et al*. (2009). Four different genotypes were detected: 1 each of type II, *Africa 1* and *Africa 2* and 2 unique strains. The *Africa 1* isolate was identified as ToxoDB#6 (ToxoDB ID EUSMPL0040-1-140). A total of 12 isolates from the Democratic Republic of Congo were genotyped which consisted of 9 type III isolates, and 1 each of type I, type II and *Africa 1*. Four of the type III isolates originating from chickens were also designated as ToxoDB#2 (Table 5). One *T. gondii* isolate was genotyped as type III, the isolate originated from the cerebral biopsy of an immunocompromised patient from the Central African Republic; this was also part of the previously mentioned study by Ajzenberg *et al*. (2009). No ToxoDB genotypes were identified from the Central African Republic.

#### Southern Africa

Only data from South Africa were retrieved (2 records, 5 datasets), which made up 7.1% (5/70) of the total number of datasets (Hammond-Aryee, 2016; Lukášová *et al*., 2018). Most isolates from South Africa were genotyped as type II (18), followed by 4 atypical strains and 1 type III strain (Table 2). No ToxoDB genotypes were identified from South African studies.

## Genetic diversity of *T. gondii* in host species

**Fig. 4.**
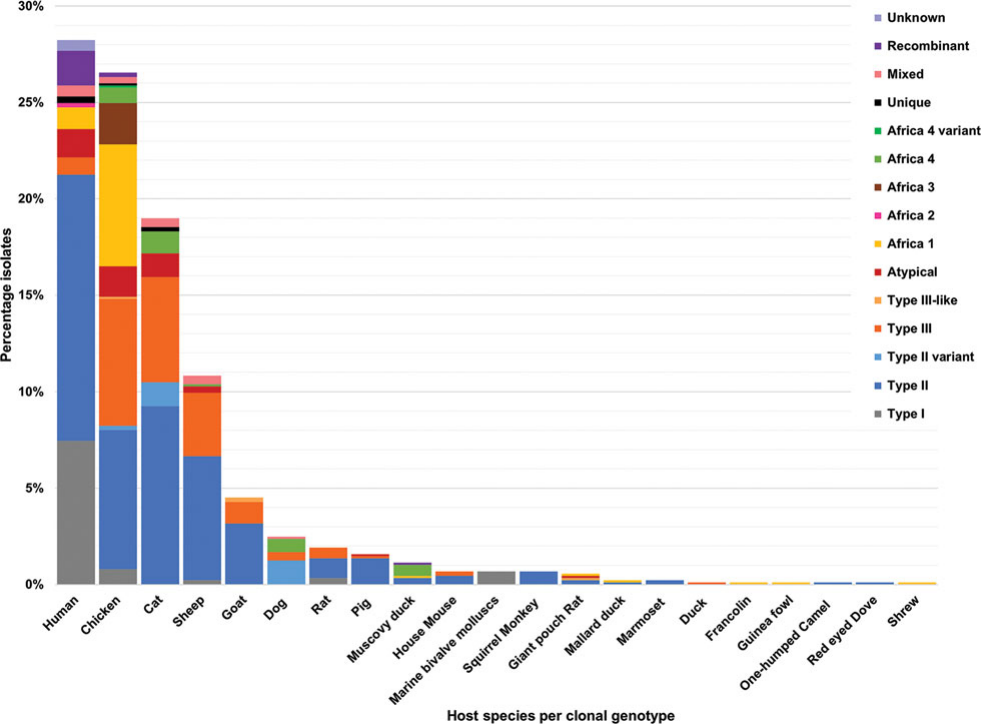
Genetic diversity of *T. gondii* clonal genotypes among host species displayed in percentages. Different colours are representative of each genotype.

**Fig. 5.**
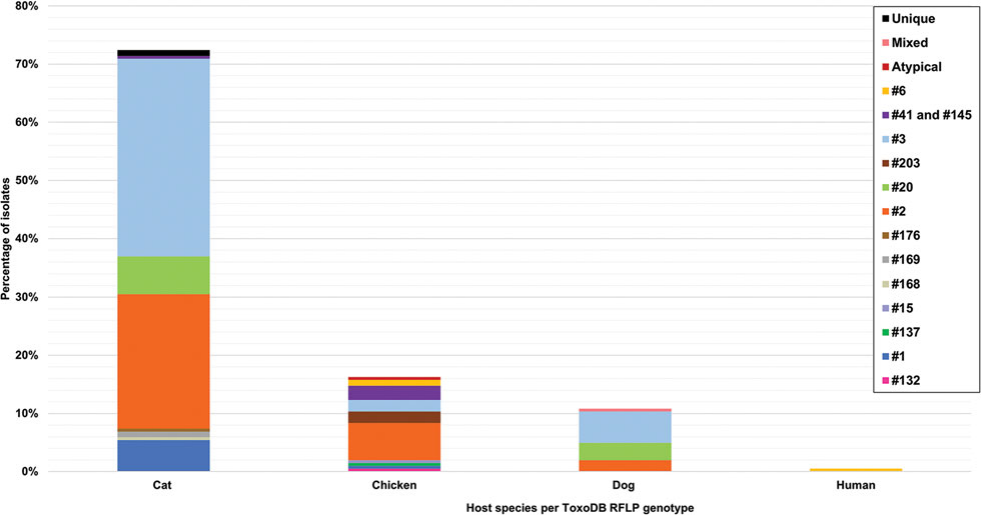
Genetic diversity of *T. gondii* ToxoDB PCR-RFLP genotypes among host species displayed in percentages. Different colours are representative of each genotype.

Clonal genotypes of *T. gondii* were identified from samples of 21 different host species. Thirteen per cent of samples and 28.2% of genotyped isolates were from humans. Eighty-nine per cent of samples and 71.8% of the genotyped isolates were from animals. In the samples from humans, a total of 250 isolates were characterized into clonal type lineages which consisted of types I, II, III, atypical, *Africa 1*, *Africa 2*, unique, mixed, recombinant and unknown genotypes (Fig. 4). The dominant clonal genotype isolated from human samples was type II. Most type I, II and recombinant genotypes were identified from human samples. *Africa 2* genotype and unknown genotypes were characterized from human samples only. Only 1 ToxoDB genotype was identified among the human samples (Fig. 5), a ToxoDB#6 isolate (EUSMPL0040-1-140) was also genotyped as *Africa 1* (Table 5). The sample originated from a Cameroonian acquired immunodeficiency syndrome patient with toxoplasmic encephalitis (Ajzenberg *et al*., 2009).

**Fig. 6.**
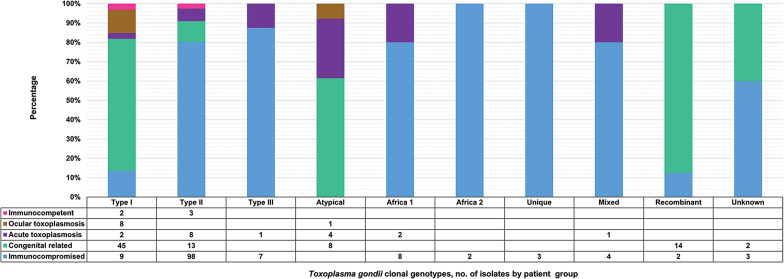
*Toxoplasma gondii* clonal genotypes isolated from human samples, grouped into patient groups according to population details. Different colours are representative of the patient groups and are displayed proportionately.

The clonal genotypes obtained from clinical samples were grouped according to the population details into 5 patient groups (Fig. 6). Isolates from the immunocompromised group were the most out of all the isolates identified from human samples (136/250). The most diversity of genotypes was found among immunocompromised individuals, with type II being the dominant genotype in this group. In contrast, type I was predominant in the congenital-related group. This group included isolates from studies where the study population consisted of pregnant women, women with terminated pregnancies and cases of congenital toxoplasmosis or infants (Boughattas *et al*., 2010, 2011*a*, 2011*b*; Tolba *et al*., 2014; Nassef *et al*., 2015; Badr *et al*., 2016; Eldeek *et al*., 2017; Lahmar *et al*., 2020; Nzelu *et al*., 2021). Type I was also the dominant genotype in cases involving ocular toxoplasmosis (Tolba *et al*., 2014; Ali *et al*., 2018). The acute toxoplasmosis group included populations with acute *T. gondii* infection, immunocompetent individuals with acute acquired toxoplasmosis and patients presenting with lymphadenopathy (Tolba *et al*., 2014; Hammond-Aryee, 2016; Leroy *et al*., 2020). Type II was mostly found in this group. In a study conducted by Ayi *et al*. (2016), in the immunocompetent group, 2 type I isolates and 3 type II isolates were identified. Atypical genotypes were exclusively identified from samples in the congenital-related, ocular and acute toxoplasmosis groups. *Africa 2* and unique genotypes were only identified in samples from immunocompromised individuals.

Among the samples tested from animal hosts, the most genotyped *T. gondii* isolates were from chickens. *Africa 4* variant and *Africa 3* were only isolated from chickens. Type II genotypes dominated among chicken samples as well as in samples from 14 other animal species (Fig. 4). Three or more different genotypes were seen in samples from chickens, cats, sheep, goats, dogs, rats, pigs, Muscovy ducks and giant pouch rats. Less diversity of *T. gondii* genotypes and fewer isolates were found in samples from house mouse, squirrel monkey, Mallard ducks, marmoset, duck (species unspecified), francolin, guinea fowl, one-humped camel, red-eyed dove and a shrew (Fig. 4).

RFLP ToxoDB genotypes were identified in samples from cats, chickens and dogs (Fig. 5). The most ToxoDB genotyped isolates were from cats (147/203) followed by chickens (33/203) and dogs (22/203). The majority of genotypes were ToxoDB#3 in dogs and cats and ToxoDB#2 in chickens. These 2 genotypes were isolated from all 3 animal hosts. ToxoDB#1 and #41/#145 were only isolated from cats and chickens. ToxoDB genotypes #168, #169, #176 and 2 unique genotypes were isolated only from cats. Genotypes only isolated from chicken samples included ToxoDB#132, #137, #15, #203, #6 and an atypical isolate. ToxoDB#20 was only present in cat and dog samples. One mixed genotype was only identified from a dog sample.

### Molecular markers

Reviews on the genetic diversity of *T. gondii* generally exclude records where less than 5 molecular markers are used for genotype identification. The reason for this is that an insufficient number of molecular markers will not detect diversity across multiple loci, thus polymorphisms present on loci other than the one targeted will not be detected and the true extent of diversity will be underestimated (Chaichan *et al*., 2017; Fernández-Escobar *et al*., 2022). In this review, less than 5 molecular markers were used in 31% (13/42) of studies which also constitute 27% (19/70) of datasets (Table 4). The most popular markers used in these studies were 3′*SAG2* and 5′*SAG2* targeting the surface antigen 2 gene. A review of *T. gondii* diversity in Europe found that 40% of the PCR-RFLP studies screened for inclusion used a single-locus method only (Fernández-Escobar *et al*., 2022). In this review, 19% (8/42) of studies used the 3′*SAG2*/5′*SAG2* combination for genotyping of the *SAG2* locus, 4.7% (2/42) used only 1 marker (*GRA6* or *SAG3*), 4.7% (2/42) used 3 markers and 1 study, by El-Alfy *et al*. (2019), used 12 markers but genotyping was only successful with 2 of the 12 markers (Table 4).

**Fig. 7.**
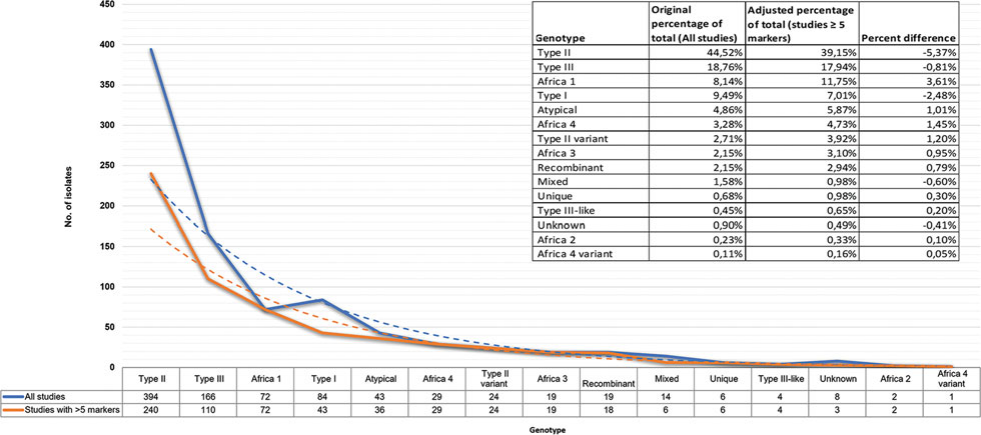
*Toxoplasma gondii* clonal genotypes identified from all studies compared to only studies that used 5 or more molecular markers.

Analysis was performed by comparing the overall diversity data of studies that used any number of molecular markers to the diversity results of studies using 5 or more molecular markers (Fig. 7). Differences were minimal, lower percentages of type I (−2.48%), type II (−5.37%), type III (−0.81%), mixed (−0.60%) and unknown (−0.41%) strains would be seen but it would not change the overall diversity pattern. Type II and III would still be the most prevalent genotypes, and type I prevalence would decrease to be slightly less than *Africa 1*. Cohen's *D* effect size (standardized mean difference) of 0.21 was calculated (Table 7). The difference between the means of the overall results, after including or excluding studies with less than 5 molecular markers, is 0.2 standard deviations, which is small and of little practical significance (Glen, 2022). All studies irrespective of the number of molecular markers were therefore included in this review.

**Table 7.** Effect size (Cohen's *D*) between all studies *vs* studies with only ≥5 markers

	No. of isolates (all studies)	Adjusted No. isolates (studies with only ≥5 markers)	Formula for Cohen's *D*: *d* *=* *(M*_1_*–M*_2_)/*s*_pooled_ *Where*: *M*_1_ = mean of group 1 *M*_2_ = mean of group 2 *s*_pooled_ = pooled standard deviations for the 2 groups. 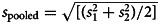 ^a^	
Mean	59	40.86666667	Difference in means	18.13333
Standard deviation (s.d.)	102.5120202	62.78178	Pooled s.d.	85.00078
Sample size	885	613	Cohen's *D*	0.213331
			Result = small effect size	

a Formula and interpretation according to Glen (2022).

The ToxoDB database was explored for isolates from African countries and isolate names from the original publications were cross-checked. All the isolates on the ToxoDB database from African countries are also present in the studies used in this review. This was a good way to screen for missing results and to confirm the sensitivity of the initial screening process. ToxoDB RFLP genotypes can also be determined by entering genotyping results for each molecular marker of the isolates into the ToxoDB ‘RFLP type’ search function. However, this only works if RFLP results are available and if 10 or more RFLP markers were used.

## Discussion

### Risk of bias in studies and reporting

There is a scarcity of information on *T. gondii* genetic diversity in African countries, especially in Southern Africa. In this review, apart from 2 studies from South Africa, no other records were found for Southern African countries. Most African studies on *T. gondii* primarily focus on prevalence and associated risk factors; molecular tests in general are an expensive commodity. Hence, in this review, studies using less than 5 molecular markers were included to avoid missing any information pertaining to the genotypes of *T. gondii* in Africa. Several isolates identified in this manner were later characterized using Mn-RFLP-PCR during the construction of the ToxoDB database (Dubey *et al*., 2003, 2005).

It should be noted that results from studies that only target the *SAG2* locus with PCR-RFLP should be treated with caution. In these instances, atypical, recombinant or exotic strains could be misclassified as type I strains (Ajzenberg *et al*., 2002; Boughattas *et al*., 2010). It is thus possible that some of the type I cases in this review (Nassef *et al*., 2015; Badr *et al*., 2016) are perhaps infected with recombinant or atypical strains or even *Africa 1* strains which also contain type I alleles. Repeat testing of these strains using either multiplex PCR of MS markers or Mn-PCR-RFLP genotyping would be advantageous. The *Africa 1* genotype like clonal type I is also highly virulent in laboratory mice (Mercier *et al*., 2010).

The high number of ToxoDB RFLP isolates from Egypt (68.97%) should be taken into consideration before concluding on the overall prevalence of *T. gondii* genotypes in Africa. An overrepresentation of strains from one geographical area will undoubtedly skew the results, which will not be representative of Africa but rather the areas where more sampling was done.

In compiling this review, 1 reviewer screened records and collected data which could introduce some risk of error. However, all search results and data were verified by 3 other reviewers. The number of databases used in this review was limited to 6, but by using well-known databases with a wide coverage, the possibility of missing major records and datasets is reduced.

### Genetic diversity and distribution

The genetic diversity of *T. gondii* in Africa has so far been described to have a mostly clonal structure, like that of North America and Europe, but with occurrences of African-specific regional or local genotypes, atypical isolates and unique ToxoDB-defined isolates (Dardé, 2008; Sibley *et al*., 2009; Galal *et al*., 2018, 2019*a*). In this review, clonal type II strains were widely distributed among the regions except in Central Africa where type III was more dominant. In West Africa, clonal type II isolates were not found in Benin, Guinea and Côte d'Ivoire; however, the *Africa 1* genotype was dominant. ToxoDB#2 and #3 amounted to similarly elevated prevalences as clonal types III and II, although ToxoDB#3 strains were mostly from North (Egypt) and East Africa (Ethiopia, Kenya). ToxoDB#1, which like ToxoDB#3 is of the type II lineage, was only present in Egypt and Ethiopia.

ToxoDB#2 (type III lineage) was more widely distributed but not found in the countries around the Gulf of Guinea (Ghana, Nigeria, Cameroon and Gabon). Similarly, a low incidence of type III clonal genotypes was found in this geographical area, apart from Gabon where a relatively high number of clonal type III isolates were identified using multiplex PCR of 13 MS markers by Mercier *et al*. (2010). If the type III isolates identified by Mercier *et al*. (2010) were to be analysed by Mn-PCR-RFLP, they would probably be classified as ToxoDB#2; however, only the *Africa 1* and *Africa 3* isolates from Gabon (identified by Mercier *et al*., 2010) were further defined as ToxoDB#6 and #203 by Shwab *et al*. (2014).

Type I clonal genotypes were found in studies from Egypt, DRC, Ghana, Libya, Tunisia and Uganda. The bulk of the type I isolates were from studies in Egypt; studies on humans were mostly related to congenital toxoplasmosis and 1 study was done on rats. The type I isolates from rats genotyped by El Fadaly *et al*. (2016) were all from *Rattus norvegicus* and were shown to be highly virulent in mouse bioassays. Wild rodent species are significant intermediate reservoirs for *T. gondii* as they are commonly preyed upon by cats and form an integral part of the life cycle (Webster, 1994; Galal *et al*., 2019*c*). The virulence effect of *T. gondii* in wild rodent species is not the same as in laboratory mice. Native rodent species in a geographical area can develop resistance to virulent *T. gondii* strains circulating within that environment (Galal *et al*., 2019*c*).

Type I isolates from Tunisia were sourced from cases of human congenital toxoplasmosis, sheep and marine bivalve molluscs. The sheep and human samples were from the same geographical area and the type I strains grouped phylogenetically with the virulent RH type I reference strain as well as type I strains from Ugandan chickens (Boughattas *et al*., 2014). Among ruminants, type I strains have been identified in samples from sheep in Spain, the UK and Iran, in samples from goats from Japan, China and Italy, in cattle from China and camels in Abu Dhabi (Sharif *et al*., 2017). Ghozzi *et al*. (2017) tested wild shellfish as bioindicators to investigate the contamination of Tunisian coastal areas with protozoan parasites. The authors attributed the high level of oocyst-contaminated clams (*Ruditapes decussatus*) to the large numbers of stray cats in Tunisia.

Free-range chickens have also been classified as good bioindicators of *T. gondii* prevalence in the environment; as they roam freely, they are more likely to ingest oocysts mixed with foodstuffs in the soil (Dubey *et al*., 2005). Samples from chickens of the DRC and Uganda harboured a small number of type I isolates (Dubey *et al*., 2005; Lindström *et al*., 2008; Bontell *et al*., 2009). Interestingly, no type I isolates were identified in a study by Lachkhem *et al*. (2021) on genotypes of *T. gondii* in chickens from Tunisia. Similarly, no type I isolates were found in chickens from Egypt by Dubey *et al*. (2003). In this review, no ToxoDB#10 genotypes (that correlates to clonal type I) were recorded among the isolates genotyped by Mn-PCR-RFLP.

The *Africa 1* genotype was identified in West- and Central African countries as well as Uganda in East Africa. The distribution seems to overlap with the countries that have tropical rainforest biomes, near the equator. ToxoDB#6 (RFLP type of *Africa 1*) was also identified only within this geographical area. This corresponds with findings in a review by Galal *et al*. (2018). The *Africa 1* genotype belongs to haplogroup 6 which also comprises several Brazilian strains. In addition, haplogroup 6 is clustered into clade A together with strains from haplogroup 1 (contains clonal type I and atypical Central/South American strains) and haplogroup 14 (consisting of *Africa 3*, equivalent to ToxoDB#203 and atypical Brazilian strains), an obvious shared ancestry has been revealed among these haplogroups (Su *et al*., 2012; Shwab *et al*., 2014). It has been proposed that type I could be a divergent strain of *Africa 1* and that this genotype might have been introduced from Africa to the Americas *via* the transatlantic slave trade between the Americas, Europe and Africa from the 16th to the 19th century (Mercier *et al*., 2010; Hamidović *et al*., 2021). Cases of infection with *Africa 1* have also been recorded in Europe and Turkey. Isolates were found in human new-borns and stray cats in Turkey, human amniotic fluid and a central nervous system sample in Denmark as well as ocular fluid samples from humans in France (Fekkar *et al*., 2011; Can *et al*., 2014; Chaichan *et al*., 2017; Jokelainen *et al*., 2018).

Mercier *et al*. (2010) suggested that *Africa 1* and *Africa 3* strains may be new major clonal lineages due to the extensive distribution of these types and the predominantly clonal propagation of *T. gondii* in the domestic environment. The authors themselves however state that more sampling is needed to confirm such a theory. In this review, *Africa 3* (ToxoDB#203) strains were isolated from chickens in several towns in Gabon. This regional genotype was found to be virulent for mice but less virulent than *Africa 1* (Mercier *et al*., 2010). Its presence only in Gabon could indicate that this strain has established itself in this country and is circulating readily within the environment.

*Africa 4* isolates and the equivalent ToxoDB#20 RFLP type isolates are present in North-, East- and West Africa, Asia and the United Arab Emirates (Dubey *et al*., 2013; El Behairy *et al*., 2013; Chaichan *et al*., 2017; Galal *et al*., 2019*b*; Lachkhem *et al*., 2021). These genotypes have been isolated from: dogs in Egypt and Sri Lanka, cats in Ethiopia and China, sand cats in the United Arab Emirates; sheep in Tunisia and a variety of hosts consisting of chickens, Muscovy ducks and a cat in Senegal. The *Africa 4* variant was isolated from a chicken in Benin and ToxoDB#137, which is the RFLP equivalent to the *Africa 4* variant, was isolated from a chicken in Ghana (Dubey *et al*., 2008; Hamidović *et al*., 2021). This genotype has also been described in Serbia from the amniotic fluid of an immunocompromised patient with no history of travel and in a congenitally infected case from Bulgaria (Uzelac *et al*., 2021).

The dispersal of African *T. gondii* genotypes to other countries could involve several pathways. The land and maritime trade between Northeast Africa, Asia and Europe *via* the Silk Road may have contributed to the dissemination of *T. gondii* genotypes (Chaichan *et al*., 2017; Galal *et al*., 2019*b*). Seasonal migration of wild birds between Africa and Europe may also play a role in the accrual of African genotypes in European countries (Uzelac *et al*., 2021). Every year 5 billion wild birds of 187 species migrate from Europe and Asia to Africa (Parin *et al*., 2018). Migratory birds can spread a variety of diseases to resident bird populations and poultry during stopovers along their flyways (Parin *et al*., 2018). It is feasible that *Toxoplasma*-infected migratory birds may en route to their destination introduce new genotypes into an environment, *via* predation or succumbing to disease and being eaten by scavengers (Uzelac *et al*., 2021).

Illegal animal smuggling and illicit animal shipments contribute massively to the distribution of zoonotic diseases across the world. Between 2009 and 2019, approximately 500 high-zoonotic-risk trafficking instances were identified in the aviation sector (Spevack, 2020). These high-risk instances involve live animals, bushmeat, domesticated animals and animal products that transit through or are destined for virtually every region across the globe (Spevack, 2020). Practices such as these can undoubtedly contribute to the proliferation of non-native *T. gondii* genotypes into a new region.

In Central/South America, the assortment of *T. gondii* genotypes could demonstrate that recombination of the parasite takes place more frequently, possibly due to less intensive agricultural breeding, the presence of a wide variety of wildlife hosts, the more frequent sexual replication within a higher number of wild felids that cover large territories and the already established presence of a large gene pool of ancient parasite lineages with more recombinations and mutations (Ajzenberg *et al*., 2004; Wendte *et al*., 2011; Su *et al*., 2012; Shwab *et al*., 2018; Galal *et al*., 2019*a*). In this review, atypical strains solely identified in Africa are ToxoDB#132, #168, #169, #176 and #203. These strains were found in cats from Egypt, and chickens in Gabon and Ghana (Dubey *et al*., 2008; Al-Kappany *et al*., 2010; Mercier *et al*., 2010). Unclassified unique *T. gondii* genotypes were isolated exclusively in samples from Cameroon, Gabon, Côte d'Ivoire and Ghana (Ajzenberg *et al*., 2009; Mercier *et al*., 2010; Pappoe *et al*., 2017). This indicates that there is a relative amount of genetic diversity different to the predominantly clonal structures seen in Europe. Most studies across the world have been focused on sampling from only humans and domestic animals, the genetic diversity of *T. gondii* isolates infecting wildlife is much greater, with more virulent isolates in the wild, which could be a public health concern as humans encroach into previously unpopulated areas (Wendte *et al*., 2011). Widespread sampling of all African countries, especially of wildlife, could perhaps reveal a variety of genotypes like Central/South America.

### Disease in humans

A high prevalence of type I strains which are more prone to reactivation was found in samples of HIV-positive patients by Khan *et al*. (2005). In contrast, Ajzenberg *et al*. (2009) found a predominance of type II isolates among 88 samples from immunocompromised individuals around the world, with a large proportion of type I/III recombinant isolates identified from African patients with reactivated chronic infections. Although a correlation between geographic origin and strain types was established, the authors concluded that there was no significant difference in the clinical indicators and the outcome when graded against the genotype involved. In this review, different genotypes were found among the immunocompromised clinical group with a dominance of type II isolates, corresponding to overall observations by Ajzenberg *et al*. (2009). Another observation was that *Africa 1* isolates were found more in the immunocompromised group than in the other clinical groups. Seeing as *Africa 1* types contain type I alleles, a possible correlation can also be formed with the findings of Khan *et al*. (2005). *Africa 2* and unique strains were only identified in the immunocompromised group and not in any of the other clinical groups. Africa 2 was also not identified among any of the other species mentioned in this review. Unique genotypes were however also identified in cats and a chicken (Mercier *et al*., 2010; Pappoe *et al*., 2017).

A possible connection between specific *T. gondii* genotype and congenital toxoplasmosis as well as ocular toxoplasmosis is seen in this review. There have been reports on severe or complicated cases of congenital toxoplasmosis linked to infections with type I, atypical and recombinant I/III or I/II strains (Boughattas *et al*., 2010, 2011*a*, 2011*b*; Delhaes *et al*., 2010; Eldeek *et al*., 2017). The main factor for the severity of congenital toxoplasmosis is the stage of pregnancy at the time of infection; infection in early pregnancy has more severe consequences than infection late in pregnancy (Ajzenberg *et al*., 2002). A review of the strain hypothesis showed that cases of congenital toxoplasmosis with atypical strains are more likely to have a poor outcome irrespective of the pregnancy trimester when compared to type II strains that cause fewer complications when the infection is acquired at the beginning of the third trimester (Ajzenberg *et al*., 2002; Delhaes *et al*., 2010; Xiao and Yolken, 2015). In addition, the activation of host susceptibility genes for psychoses has been linked specifically to type I strains, with the adult offspring of type I infected mothers having an increased risk for the development of psychotic illnesses and schizophrenia (Xiao *et al*., 2009).

On analysis of clinical manifestations in humans, most *T. gondii* type I strains in this review were from samples of congenital-related toxoplasmosis cases; these type I isolates were exclusively from Tunisia and Egypt. Other genotypes in this clinical group were type II, recombinant types I/III and I/II, atypical genotypes and unknown types. Most studies were on women that had abnormal or severe pregnancy outcomes (Boughattas *et al*., 2010, 2011*a*, 2011*b*; Nassef *et al*., 2015; Badr *et al*., 2016; Eldeek *et al*., 2017). In all but one of the studies in this review, the congenital cases related to type I infections had severe outcomes. Patients in the study by Tolba *et al*. (2014) were asymptomatic, despite evidence of type I and atypical genotypes of *T. gondii* in the samples. Another Egyptian study on females with adverse pregnancy outcomes stated in their abstract that *T. gondii* type II was found more (87%) than type I (13%) (Abdel-Hameed and Hassanein, 2008). This record was not recovered during the compiling of articles for this review and only discovered later in the review process; full text for this publication could not be retrieved upon an investigation of this omission. Type I, II and unknown genotypes were detected from samples of women infected in their first trimester that presented with terminated pregnancies (Badr *et al*., 2016). The higher prevalence of type I genotypes in this review and the detection of type II in first-trimester-infected cases, having adverse outcomes in both instances, correlate with the findings of Rico-Torres *et al*. (2016) and Ajzenberg *et al*. (2002). Type I and atypical isolates were linked to severe congenital toxoplasmosis. Moreover, type I as well as type II are implicated in lethal infections of untreated congenital toxoplasmosis and maternal infections with type II during the first trimester were more likely to lead to fetal damage.

In Lahmar *et al*. (2020), Tunisian women acquired infection with type II during pregnancy, 1 infant out of 4 presented with peripheral chorioretinitis scarring at 1-month follow-up. In the 3 asymptomatic infants, the mothers became infected late in the second trimester. Type II strains cause fewer complications when infection is acquired late in the pregnancy (Ajzenberg *et al*., 2002; Xiao and Yolken, 2015). Even though exposed infants are asymptomatic after birth, there are risks for complications later in life from non-treatment, such as brain calcifications, hydrocephalus, ocular disease or psychoses (Xiao and Yolken, 2015; Lahmar *et al*., 2020). In a Nigerian study on *T. gondii* seropositive pregnant women, the authors found type II and atypical strains that shared haplotypes with isolates from samples of free-range chickens and/or pigs collected in the same study areas (Nzelu *et al*., 2021). This highlights the importance of using genotyping in epidemiological studies and determining infection sources.

Recombinant (I/III and I/II) and atypical genotypes pose a great threat regarding the severity of congenital toxoplasmosis (Ajzenberg *et al*., 2002; Delhaes *et al*., 2010; Xiao and Yolken, 2015). A *T. gondii* I/III recombinant strain led to the demise of an infected preterm infant diagnosed with chorioretinitis and cardiopathy (Boughattas *et al*., 2011*b*). In a different study by Boughattas *et al*. (2010), recombinant strains I/III and I/II as well as mixed infections with both I/II and I/III were detected in congenital toxoplasmosis cases in Tunisia. Unfortunately, the clinical outcomes could only be established in 3 of the cases, 1 neonate developed chorioretinitis and 2 had no toxoplasmic symptoms. A Tunisian case study on a diabetic woman with first-trimester toxoplasmosis discovered an atypical *T. gondii* strain. Multilocus genotyping of the isolate confirmed a new genotype with an uncommon pattern of type I, II, III and non-archetypal alleles (Boughattas *et al*., 2011*a*). Due to the gestational age and the increased risk of severe damage to the fetus from such atypical strains, the pregnancy was terminated. Similarly, Delhaes *et al*. (2010) reported on a congenital toxoplasmosis case from a French woman infected with an atypical multilocus genotype. Despite proper management with anti-*Toxoplasma* treatments, bilateral ventricular enlargement and calcifications developed in the fetus; subsequently, this pregnancy was terminated.

*Africa 1* strains have been isolated from CSF samples of new borns with congenital toxoplasmosis in Turkey by Döşkaya *et al*. (2013); none of the *Africa 1* strains in this review was from congenital-related samples. *Africa 1* was only found in samples from immunocompromised patients and immunocompetent individuals with acute acquired toxoplasmosis (Ajzenberg *et al*., 2009; Leroy *et al*., 2020). ToxoDB#41 was identified among the genotypes in congenital toxoplasmosis neonates from southeast Brazil (Carneiro *et al*., 2013). These neonates presented with retinochoroidal lesions. Interestingly, similar strains were isolated from samples of chickens and cats in Ghana. They were characterized as both ToxoDB#41 and #145; these types have also been found in chickens and opossums in Brazil. No ToxoDB genotypes were characterized among the congenital related infections in this review. An opportunity thus arises for the development of surveillance programmes for *T. gondii* genotypes linked to congenital toxoplasmosis in African countries, to find relationships between genotypes in Africa and those in other continents and to aid in tracking sources of infection such as food animals.

Healthy people can develop ocular toxoplasmosis, and choroiditis may lead to permanent loss of vision (Hammond-Aryee *et al*., 2014; Despommier *et al*., 2019). Ocular toxoplasmosis has been frequently associated with infection by type I, atypical and recombinant strains (Khan *et al*., 2005; Dardé, 2008; Tolba *et al*., 2014; Xiao and Yolken, 2015; Ali *et al*., 2018; Pomares *et al*., 2018). In the ocular toxoplasmosis group in this review, apart from 1 atypical isolate, all other strains were type I (Tolba *et al*., 2014; Ali *et al*., 2018).

Severe and fatal cases of visceral toxoplasmosis, from atypical strains, have been reported in immunocompetent individuals. A highly pathogenic Amazonian strain has been associated with numerous cases of disseminated toxoplasmosis in immunocompetent individuals in French Guiana (Carme *et al*., 2002). An outbreak from a single atypical strain caused mild illness in some individuals and serious illness or death in others (Demar *et al*., 2007). In the acute toxoplasmosis clinical group of this review, type I, II, III, atypical, *Africa 1* and mixed genotypes were identified. The presence of atypical isolates in this clinical group and the absence of such isolates among the immunocompetent group do indicate a degree of involvement. Only type I and type II strains were identified in the immunocompetent group in this review.

## Conclusion

Determining which genotypes of *T. gondii* are prevalent in Africa can aid in its prevention and control. It is clear from this review that connections exist between specific *T. gondii* genotypes, disease manifestations and severity as well as geographic locale. There is however a scarcity of information on *T. gondii* in Africa, especially in Southern Africa. Future studies should not only focus in determining the prevalence of *T. gondii* infection in Africa but also on the genotypes involved. Uniform genotyping methods need to be employed with a continent-wide sampling of an extensive host range involving humans, domestic animals and wildlife. One would only then be able to form a clear concept of the diversity of *T. gondii* in Africa.

## Data Availability

A protocol of this systematic review with supplementary data on the search results and data analysis is registered on the Open Science Framework (OSF, https://www.cos.io/). The registration link is: https://doi.org/10.17605/OSF.IO/VM4GS and the project data are available at https://osf.io/gxkst.

## Supplementary material

For supplementary material accompanying this paper visit https://doi.org/10.1017/S0031182023000252. click here to view supplementary material
