# Supplementary material for: An African perspective on the genetic diversity of Toxoplasma gondii: A systematic review
Source: Parasitology. 2023 Mar 20;150(7):551–78. doi: 10.1017/S0031182023000252 (PMC10260301; doi:10.1017/S0031182023000252)
Supplement: Supplementary file 1 [file S0031182023000252sup001.pdf]

Table S1. Details of search strategies used in six online databases

Source	Search interface/software	Query string	Records	Query Date	year first	year last
**Scopus**	https://www.scopus.com	(ALL(Africa) AND TITLE-ABS-KEY(toxoplasmosis) OR TITLE-ABS-KEY(toxoplasma) AND TITLE-ABS-KEY(genotype) OR TITLE-ABS-KEY(genetic) OR TITLE-ABS-KEY(diversity) OR TITLE-ABS-KEY(molecular) OR TITLE-ABS-KEY(strain*) OR TITLE-ABS-KEY(pcr) OR TITLE-ABS-KEY(markers)) AND (LIMIT-TO (DOCTYPE,"ar")) AND (LIMIT-TO (LANGUAGE,"English"))	415	2022/04/03	1987	2022
**Europe PMC**	www.europepmc.org	(("Africa") AND (ABSTRACT:"toxoplasma" OR ABSTRACT:"toxoplasmosis") AND (ABSTRACT:"genotype" OR ABSTRACT:"genetic" OR ABSTRACT:"diversity" OR ABSTRACT:"molecular" OR ABSTRACT:"strain*" OR ABSTRACT:"pcr" OR ABSTRACT:"markers") OR (KW:"toxoplasmosis" OR KW:"toxoplasma" OR KW:"genotype" OR KW:"genetic" OR KW:"diversity" OR KW:"molecular" OR KW:"strain*" OR KW:"pcr" OR KW:"markers") AND (LANG:"eng" OR LANG:"en" OR LANG:"us")) AND (((SRC:MED OR SRC:PMC OR SRC:AGR OR SRC:CBA) NOT (PUB_TYPE:"Review")))	439	2022/04/03	1969	2022
**Google Scholar**	Harzings Publish or Perish (software) https://harzing.com/resources/publish-or-perish	(Africa) AND (toxoplasma OR toxoplasmosis) AND (genotype OR genetic OR diversity OR molecular OR strain OR pcr OR markers) **Limit to first 1000 relevant results**	990	2022/04/03	1915	2022
**Pubmed**	https://pubmed.ncbi.nlm.nih.gov/	**Search: Africa AND (toxoplasma OR toxoplasmosis) AND (genotype OR genetic OR diversity OR molecular OR strain OR pcr OR markers)** ("africa"[MeSH Terms] OR "africa"[All Fields] OR "africa s"[All Fields] OR "africas"[All Fields]) AND ("toxoplasma"[MeSH Terms] OR "toxoplasma"[All Fields] OR "toxoplasmas"[All Fields] OR ("toxoplasmosis"[MeSH Terms] OR "toxoplasmosis"[All Fields] OR "toxoplasmoses"[All Fields])) AND ("genotype"[MeSH Terms] OR "genotype"[All Fields] OR "genotypes"[All Fields] OR "genotypic"[All Fields] OR "genotype s"[All Fields] OR "genotyped"[All Fields] OR "genotyper"[All Fields] OR "genotypical"[All Fields] OR "genotypically"[All Fields] OR "genotyping"[All Fields] OR "genotypings"[All Fields] OR "genotypization"[All Fields] OR ("genetic therapy"[MeSH Terms] OR ("genetic"[All Fields] AND "therapy"[All Fields]) OR "genetic therapy"[All Fields] OR "genetic"[All Fields] OR "genetical"[All Fields] OR "genetically"[All Fields] OR "genetics"[MeSH Subheading] OR "genetics"[All Fields] OR "genetics"[MeSH Terms]) OR ("diverse"[All Fields] OR "diversely"[All Fields] OR "diversities"[All Fields] OR "diversity"[All Fields]) OR ("molecular"[All Fields] OR "moleculars"[All Fields]) OR ("sprains and strains"[MeSH Terms] OR ("sprains"[All Fields] AND "strains"[All Fields]) OR "sprains and strains"[All Fields] OR "strain"[All Fields] OR "strains"[All Fields] OR "strain s"[All Fields]) OR "pcr"[All Fields] OR ("biomarkers"[MeSH Terms] OR "biomarkers"[All Fields] OR "marker"[All Fields] OR "markers"[All Fields]))	214	2022/04/03	1970	2022
**ScienceDirect**	https://www.sciencedirect.com	**Articles with terms:** Africa AND toxoplasma AND (genotype OR genetic OR diversity OR molecular OR strain OR pcr OR markers)<br>**Title, Abstract or author-specified Keywords:**(toxoplasmosis OR toxoplasma) AND (genotype OR genetic OR diversity OR molecular OR strain OR pcr OR markers) **Filter:** Research articles only	209	2022/04/03	1997	2022
**Dimensions AI**	https://app.dimensions.ai/	**Title and Abstract in Freetext:** Africa AND (toxoplasma OR toxoplasmosis) AND (genotype OR genetic OR diversity OR molecular OR strain OR pcr OR markers) **Filter publication type:** Limit to Article	90	2022/04/03	1987	2022
**TOTAL**			**2357**			
